# Breast Cancer Research to Support Evidence-Based Medicine in Nigeria: A Review of the Literature

**DOI:** 10.1200/GO.20.00541

**Published:** 2021-03-15

**Authors:** Omolara A. Fatiregun, Temiloluwa Oluokun, Nwamaka N. Lasebikan, Emmanuella Nwachukwu, Abiola A. Ibraheem, Olufunmilayo Olopade

**Affiliations:** ^1^ Department of Radiology, Oncology Unit, Lagos State University College of Medicine, Lagos, Nigeria; ^2^ University of Liverpool School of Medicine, Liverpool, United Kingdom; ^3^ Department of Radiation Medicine, University of Nigeria Teaching Hospital, Enugu, Nigeria; ^4^ Department of Radiotherapy and Oncology, National Hospital Abuja, Abuja, Nigeria; ^5^ Department of Medicine, University of Chicago, Chicago, IL; ^6^ Centre for Clinical Cancer Genetics and Global Health, University of Chicago, Chicago, IL

## Abstract

**PURPOSE:**

Breast cancer is the most common malignancy in women worldwide. In Nigeria, it accounts for 22.7% of all new cancer cases among women. Evidence-based medicine (EBM) entails using the results from healthcare research to enhance the clinical decision-making process and develop evidence-based treatment guidelines. Level 1 and 2 studies, such as randomized controlled trials, meta-analyses, and systematic reviews of randomized controlled trials, yield more robust types of evidence. This study reviewed the levels of evidence of breast cancer publications in Nigeria.

**METHODS:**

We conducted an electronic literature search of all studies published on breast cancer in Nigeria from January 1961 to August 2019. We reviewed all the articles found under the search term “Breast Cancer in Nigeria” on medical databases.

**RESULTS:**

Our search identified 2,242 publications. One thousand two hundred fifty duplicates were removed, and 520 were excluded. A total of 472 articles were considered eligible for this review. Most of these articles were case series or reports (30.7%), qualitative studies (15.7%), followed by cross-sectional studies (13.3%), laboratory studies (12.9%), case-control studies (6.1%), case reports (7%), and cohort (5.7%).

**CONCLUSION:**

Breast cancer research in Nigeria is yet to produce much evidence of the types considered to best support EBM. The scarcity of data hampers the implementation of EBM in Nigeria. Currently, most treatment guidelines are adapted from those developed in other countries, despite genetic differences among populations and different environmental influencing factors.

## INTRODUCTION

Breast cancer is the most common malignancy in women. Each year, 2.1 million women are affected. It is also responsible for 15% of all cancer-related deaths among women. More than 620,000 women died of breast cancer in 2018 alone.^1^ Although breast cancer is more prevalent in higher and upper-middle-income countries,^2^ disease rates are rising globally.^1^ In Nigeria, a lower-middle-income country, breast cancer is the most common malignancy among women, accounting for 22.7% of all new cancer cases.^3^ With 12,000 deaths in 2018, it also has the highest breast cancer mortality rate of all nations.

**CONTEXT**

**Key Objective**
Evidence-based medicine (EBM) is the key to developing treatment protocols for patients with breast cancer worldwide. There is, however, limited knowledge on the availability of studies on breast cancer in Nigeria needed to ensure EBM, especially with regard to their methods and study design. This study was performed to identify the levels of evidence of breast cancer research.
**Knowledge Generated**
Most available breast cancer studies are still at a low level of evidence (cross-sectional and case report studies). Second, despite the increasing number of breast cancer publications over the years, there are a minimal number of high-level evidence studies, randomized controlled trials, focusing on breast cancer management in Nigeria.
**Relevance**
There is a need to increase EBM levels in breast cancer research to address global disparities. This study can serve as an advocacy tool to drive governmental policies on increasing cancer research funding in Nigeria.

Several disparities have been documented between women of African descent and Caucasians. The presentation of breast cancer in the Nigerian population is characterized by more advanced, triple-negative cases than are people of European descent.^4^ In certain parts of Africa, more than 70% of women with breast cancer present at stage 3 or 4, whereas in Europe, this figure is < 50%.^5^ There is also evidence to suggest that, in sub-Saharan Africa, breast cancer occurs more frequently in premenopausal women and younger age groups than in Western nations. In terms of mortality, the disease heavily burdens the African region, with Nigeria having the highest age-standardized mortality rate.^5^

Evidence-based medicine (EBM) in clinical practice is about using the results from well-conducted healthcare research to enhance the clinical decision-making process. The EBM approach assumes that clinicians can access and use the relevant literature so that best practice can be ascertained and implemented.^6^ Meta-analyses, systematic reviews, and randomized controlled trials (RCTs) are considered to yield the most robust types of evidence and thus are recommended over case-control and cross-sectional studies as the basis for EBM. In addition to its use of the clinical practice, EBM has also been used to inform evidence-based practice policies and guidelines designed to affect both individual patients and populations.^7^

A key aspect of EBM is the ranking system used to classify levels of evidence. The Canadian Task Force first described these as part of the Periodic Health Examination in 1979.^8^ In 1989, Sackett expanded upon them in an article on levels of evidence on research on antithrombotic agents. Since then, multiple organizations have developed their classification systems,^8^ such as Essential Evidence Plus, the American Society for Plastic Surgeons, and Oxford Centre for Evidence-based Medicine. These systems specify hierarchies that place systematic reviews of RCTs and RCTs at the highest level because such studies minimize systematic bias and biases of other types. Case series and studies based on expert opinion are placed at a lower level because they are vulnerable to a variety of biases. A case series study design, for example, does not control for confounding factors.

Guidelines and treatment pathways based on the best scientifically available evidence^9^ have been developed for various malignancies, further advancing the quality of cancer care. The treatment of breast cancer, like all other malignant diseases, requires safe and effective measures that have been proven to meet high clinical standards.^10^

In certain countries, expert panels systematically review studies and trials. They develop recommendations for clinical practice.^10^ An example of this is the US Preventive Services Task Force (USPTF). In 2009, the USPTF changed its recommendations to advise against routine mammography screening before age 50, instead advocating for two-yearly mammograms between 50 and 74 years of age.^11^ This change was partly motivated by systematic reviews of RCTs that included data from other countries such as the United Kingdom and Sweden.^12^ A recent study evaluated the clinical improvement of patients with breast cancer treated according to national guidelines, compared with those who were not. The results showed that women who received treatment that 100% adhered to guidelines had significantly better clinical outcomes.^9^

Cancer diagnostic practices and therapeutic measures used in the treatment of breast cancer in Nigeria should be only those proven to be effective by high-quality research if we are to improve clinical outcomes. There have been a few attempts at developing local protocols for breast cancer management, and these are also adapted from the international guidelines like the NCCN guidelines because of paucity of high-level evidence-based studies.^13^ General awareness of EBM in Nigeria is low, and the concept of EBM has not been integrated into the healthcare system in Nigeria.^14^ Published studies on the levels of evidence achieved in Nigerian breast cancer research are few. Our study begins to address these issues by documenting the levels of evidence in published studies of breast cancer in Nigeria, as well as the availability of tools needed to ensure EBM in Nigeria.

## METHODS

**FIG 1:**
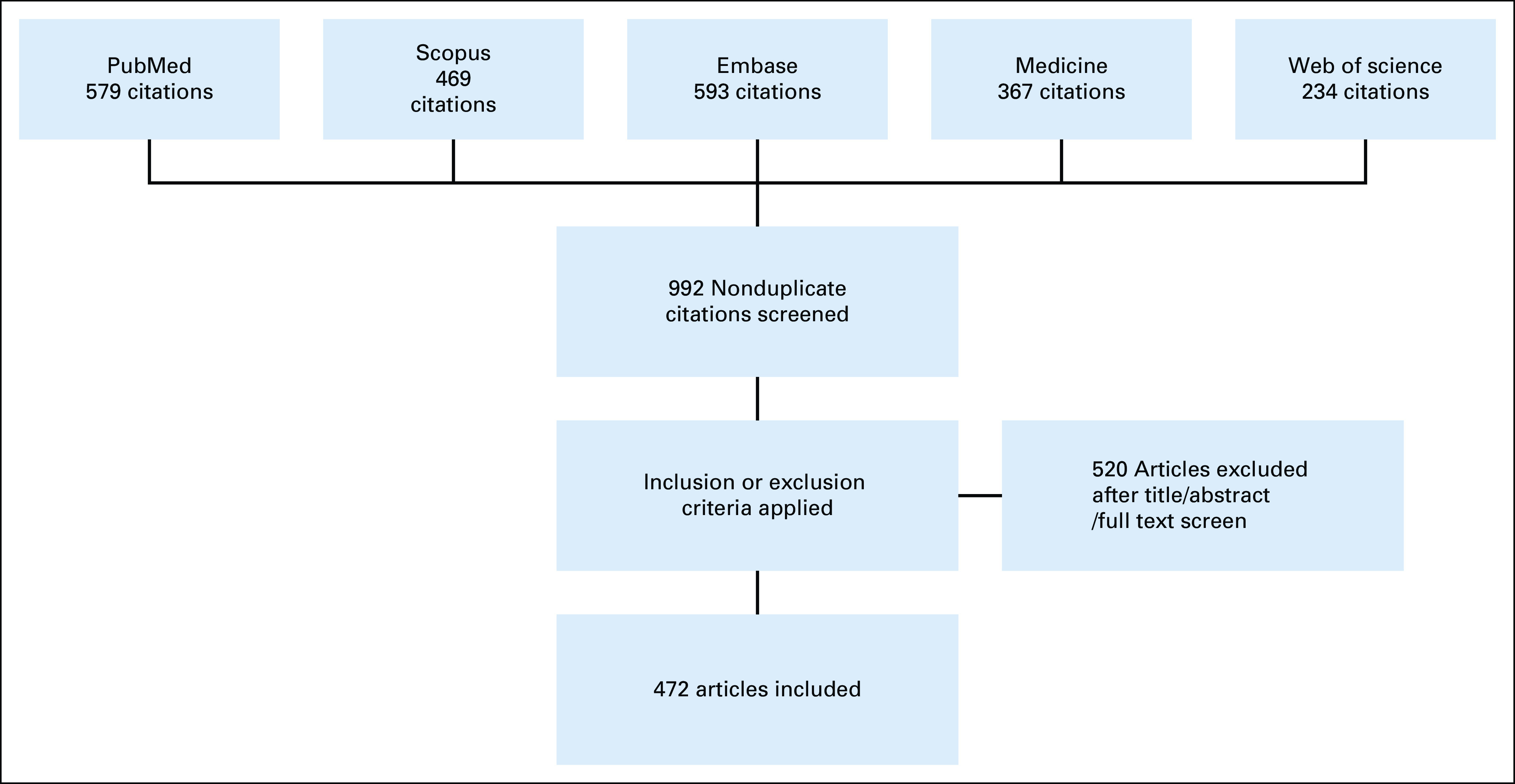
Article selection using PRISMA selection criteria. PRISMA, Preferred Reporting Items for Systematic Reviews and Meta-Analyses.

An electronic literature search of all studies published on breast cancer in Nigeria from January 1961 to August 2019 was conducted. After reviewing all the articles found under the search term “Breast Cancer in Nigeria” on medical databases such as PubMed, Scopus, Embase, Medline, and Web of Science, we categorized them based on their study designs into various levels of evidence. We organized our effort in accord with the Preferred Reporting Items for Systematic Reviews and Meta-Analyses^15^ guidelines (Fig 1) and the National Institutes of Health (NIH) guidelines.^16^

Articles were included in this review only if they had adequate information on breast cancer in Nigeria. We fulfilled this inclusion criterion based on the titles, abstracts, or full text of articles. Other data retrieved from the studies meeting our inclusion criteria included the year of publication, study design, and level of evidence according to the Oxford Centre for Evidence-Based Medicine (CEBM) classification system.^17^ The included articles were grouped into one of the five main levels of evidence.

## RESULTS

**FIG 2:**
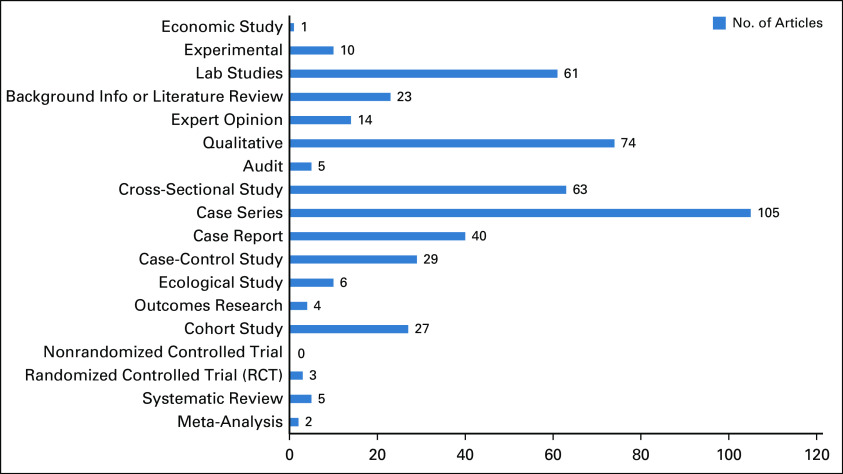
Types of study designs in Nigerian breast cancer research.

Our search yielded 2,242 publications. One thousand two hundred fifty duplicates were removed, and 520 were excluded as they did not fulfill the inclusion criteria of the study (Fig 1). A total of 472 articles were considered eligible for this review. Most of these articles were case series or reports (30.7%) and qualitative studies (15.7%), followed by cross-sectional studies (13.3%), laboratory studies (12.9%), case-control studies (6.1%), case reports (7%), cohort studies (5.7%), literature reviews or background information (4.9%), and expert opinion (3%). Ten or fewer studies in each of the categories, ecological, economic, or experimental, were found to be acceptable for this review (Fig 2). This was also the case for outcomes research, audits, systematic reviews, meta-analyses, and RCTs.

**FIG 3:**
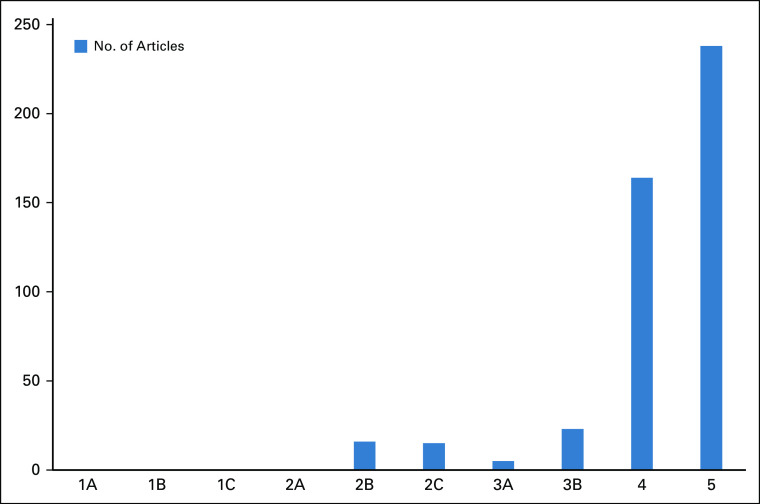
Levels of evidence in Nigerian breast cancer research.

Based on the Oxford CEBM Hierarchy of Evidence,^17^ a slight majority of studies (50.4%) were at level 5 (Fig 3). These included laboratory reports, expert opinion studies, literature reviews, ecological and economic studies, experimental studies, qualitative studies, cross-sectional studies, and audits. 34.7% of studies were at level 4 of evidence, including case reports, case series, and poor-quality cohort or case-control studies. 4.8% of the studies were at level 2b, which included individual cohort studies, and 3.2% at level 2c, which were mostly ecological, whereas level 3a of evidence constituted 1% of all selected studies. There were no studies at level 1 (a, b, or c) or 2a (systematic reviews with homogeneity of randomized clinical trials, individual randomized clinical trials, all or none of the studies, and systematic reviews of cohort studies).

**FIG 4:**
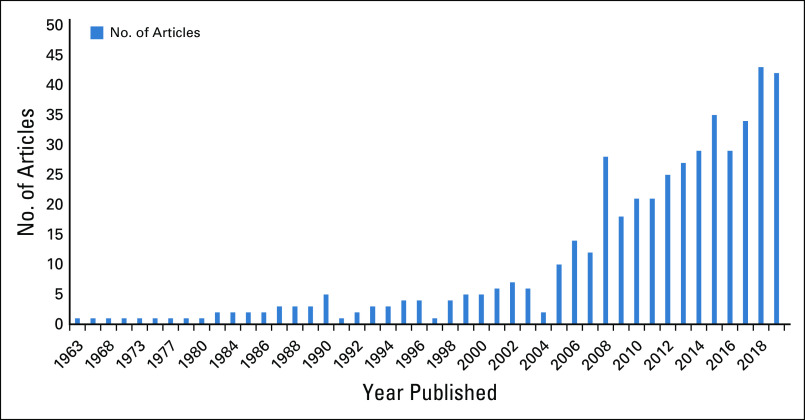
Years of publication of Nigerian breast cancer research.

Most studies were published in 2018 (43 articles were identified that year), closely followed by 2019 (42 articles). 2015, 2016, and 2017 had 35, 29, and 34 publications, respectively. There were no recorded publications in the years 1964, 1970-1972, 1975-1976, 1978, or 1981-1982 (Fig 4).

## DISCUSSION

We found that case series and reports have been the most common study designs and most of the evidence accrued is at the lowest level, level 5. Thus, this study highlights a lack of the high-quality research on breast cancer needed to foster EBM in Nigeria. Case reports and case series are descriptive studies that focus on the clinical outcomes or prognosis of one or more patients.^18^ One of the main advantages of these studies is that they are inexpensive to conduct. This may explain the frequency of their use in low-resource settings. They can also be used to identify novel therapies or rare complications of diseases.^19^ Conversely, since case series and reports lack control groups, they cannot be used to effectively compare outcomes with those of patients not having the treatment or condition under study. In the case of retrospective case series, the quality of the study is dependent on data availability and the accuracy of previous records. These are often poor in Nigeria and other developing countries.^20^ Also, this type of study design is highly subject to bias. This reduces generalizability to other populations.

In Nigeria, local and sustainable research generally remains scarce. Few level 1 and 2 studies on breast cancer have been carried out.^21^ Level 1 studies include systematic reviews of RCTs, RCTs, and all or none of the RCTs.^22^ The RCT is widely considered the gold standard of EBM and a very powerful tool in clinical research.^23^

This study, however, showed a sharp increase in the number of articles published from 2000 onward. The largest number of publications appeared in 2018. In the same year, there were 43 unique articles on breast cancer in Nigeria indexed by PubMed. Compared with countries like Kenya and Ethiopia (with fewer than 30 breast cancer studies published in each in 2018), Nigeria fared well. In contrast, the gap widens in the opposite direction when Nigeria’s research output is compared with that of more developed countries. For example, the output from the United States and the United Kingdom was well over 1,000 articles each in 2018. Erhabor et al^24^ stated that barriers such as loss to follow up, poor health infrastructure, inadequate data management, and cost-related challenges might limit the capacity to conduct RCTs in Nigeria. A report by the Global Forum for Health Research stated the importance of improving research capacity to improve the quality of health care delivered in developing countries.^25^ In Nigeria, the available research infrastructure remains inadequate at both institutional and national levels, with a shortage of skilled healthcare personnel, including statisticians, epidemiologists, health policy analysts, economists, and managers.^26^

Another major reason for the low number of studies on breast cancer in Nigeria is lack of funding. In most developed countries, the bulk of research funding comes from the government, pharmaceutical companies, and charitable foundations. Of all malignancies, breast cancer generally receives the most funding per case.^27^ In 2015, the US NIH spent $674 million in US dollars on breast cancer research.^28^ In 2018/19, the United Kingdom’s National Cancer Research Institute (NCRI) partner organizations spent £54 million in Euros on research projects studying breast cancer.^29^

In many African countries, funding for clinical research is mostly obtained from collaborations with partners in Western countries and local pharmaceutical companies.^24^ There were concerns that this could reduce the capacity of African scientists to conduct sustainable research relevant to their various countries.^26^

In Nigeria, there have been a few efforts to conduct high-level breast cancer research, often headed by research consortia with specific focus areas such as The Women of African Ancestry Breast Cancer Consortium^21^ and The Nigerian Breast Cancer Study (NCBS). Other research consortia, such as the Men of African Descent and Carcinoma of the Prostate (MADCaP) and the Prostate Cancer Transatlantic Consortium (CapTC), are examples of capacity building programs run in collaboration with researchers from developed countries.^30,31^ In Nigeria, there is little government funding for cancer research, as limited resources are predominantly allocated toward program implementation.^24^ However, this only increases the need for a reliable evidence base, to evaluate program outcomes and to ensure that resources are used efficiently.^32^ Since 2011, the main government funding channel for research has been the Tertiary Education Trust Fund.^33^ Despite an endorsement to spend 1% of their gross domestic product on research and development, a 2010 report showed that few African countries were able to meet this threshold.^26^

In conclusion, breast cancer research is currently at low levels of evidence in Nigeria. Breast cancer researchers should aim to conduct more level 1 and 2 studies, such as RCTs, meta-analyses, and systematic reviews of RCTs. Such studies will provide data that are socioculturally appropriate and economically feasible for Nigeria, leading to evidence-based treatment guidelines, which will improve the quality of breast cancer care in Nigeria.^24^ It is essential for the Nigerian government to increase funding for cancer-related research and formulate policies that aim to encourage the practice of EBM. Increased funding from philanthropic donors and corporate organizations will help to build sustainable breast cancer research infrastructure in Nigeria. Viable policies aimed at fostering high-level cancer research through government grants should also be implemented. Finally, multidisciplinary and international collaborations on breast cancer research are required to strengthen networks and address knowledge gaps.

## References

[1] World Health Organization: WHO | Breast Cancer. WHO, 2019

[2] Bellanger M Zeinomar N Tehranifar P et al: Are global breast cancer incidence and mortality patterns related to country-specific economic development and prevention strategies? J Glob Oncol 4:1-16, 2018 10.1200/JGO.17.00207 PMC6223528 30085889

[3] Globacan 2018 Nigeria Cancer Fact Sheet. https://gco.iarc.fr/today/data/factsheets/populations/566-nigeria-fact-sheets.pdf

[4] Zheng Y Walsh T Gulsuner S et al: Inherited breast cancer in Nigerian women. J Clin Oncol 36:2820-2825, 2018 30130155 10.1200/JCO.2018.78.3977 PMC6161833

[5] Azubuike SO Muirhead C Hayes L et al: Rising global burden of breast cancer: The case of sub-Saharan Africa (with emphasis on Nigeria) and implications for regional development: A review. World J Surg Oncol 16:63, 2018 29566711 10.1186/s12957-018-1345-2 PMC5863808

[6] Guyatt G: Evidence-based medicine. JAMA 268:2420-2425, 1992 1404801 10.1001/jama.1992.03490170092032

[7] Eddy D: Practice policies: Where do they come from? JAMA 263:1265-1275, 1990 2304243 10.1001/jama.263.9.1265

[8] Burns PB Rohrich RJ Chung KC: The levels of evidence and their role in evidence-based medicine. Plast Reconstr Surg 128:305-310, 2011 21701348 10.1097/PRS.0b013e318219c171 PMC3124652

[9] Montero AJ: Guidelines are essential to improving clinical outcomes in breast cancer patients. Breast Cancer Res Treat 153:1-2, 2015 26245162 10.1007/s10549-015-3526-9

[10] Beuth J: Evidence-based complementary medicine in breast cancer therapy. Breast Care (Basel) 4:8-12, 2009. 20877679 10.1159/000194306 PMC2942012

[11] Barclay L: USPSTF Issues New Breast Cancer Screening Guidelines. Medscape. https://www.medscape.com/viewarticle/712473, 2009

[12] Kerlikowske K: Evidence-based breast cancer prevention: The importance of individual risk. Ann Intern Med 151:750-752, 2010 10.7326/0003-4819-151-10-200911170-00012 19920276

[13] Lasebikan NN: University of Nigeria Teaching Hospital Breast Cancer Management Protocol (ed 1). https://www.slideshare.net/AmakaLasebikan/unth-breast-cancer-management-protocol, 2017

[14] Nwagwu W: Levels of consciousness and awareness about evidence-based medicine among consultants in tertiary health care institutions in Nigeria. Health Info Libr J 25:278-287, 2008 19076674 10.1111/j.1471-1842.2008.00768.x

[15] Liberati A Altman DG Tetzlaff J et al: The PRISMA statement for reporting systematic reviews and meta-analyses of studies that evaluate health care interventions: Explanation and elaboration. J Clin Epidemiol 62:e1-e34, 2009 19631507 10.1016/j.jclinepi.2009.06.006

[16] Systematic Review Service: NIH Library. https://www.nihlibrary.nih.gov/services/systematic-review-service

[17] OCEBM Levels of Evidence Working Group, Howick J Chalmers I et al: The Oxford Levels of Evidence 2; 1(version):5653. Oxford, United Kingdom, Oxford Centre for Evidence-Based Medicine, 2011. https://www.cebm.net/index.aspx?o=5653

[18] Parab S Bhalerao S: Study designs. Int J Ayurveda Res 1:128-131, 2020 10.4103/0974-7788.64406 PMC2924977 20814529

[19] Types of Studies, Their Advantages and Disadvantages | Deranged Physiology. https://derangedphysiology.com/main/required-reading/statistics-and-interpretation-evidence/Chapter 1.0.3/types-studies-their-advantages-and-disadvantages, 2020

[20] Evidence Based Practice: Case Reports and Case Series. https://www.caresearch.com.au/Caresearch/Portals/0/Documents/PROFESSIONAL-GROUPS/Nurses Hub/NH_EBP_CaseReports_May2012.pdf, 2020

[21] Morhason-Bello IO Odedina F Rebbeck TR et al: Challenges and opportunities in cancer control in Africa: A perspective from the African Organisation for Research and Training in Cancer. Lancet Oncol 14:e142-e151, 2013 23561745 10.1016/S1470-2045(12)70482-5

[22] Phillips B Ball C Sackett D et al: Oxford Centre for Evidence-Based Medicine Levels of Evidence. https://www.cebm.ox.ac.uk/resources/levels-of-evidence/oxford-centre-for-evidence-based-medicine-levels-of-evidence-march-2009, 2001

[23] Alex Yartsev: Advantages and disadvantages of randomised control study design. Deranged Physiology. https://derangedphysiology.com/main/cicm-primary-exam/required-reading/research-methods-and-statistics/Chapter 2.0.2/advantages-and-disadvantages-randomised-control-study-design, 2020

[24] Erhabor O Udomah F Abdulrahaman Y et al: Randomized clinical trials on breast cancer in Nigeria and other developing countries: Challenges and constraints, in Perioperative Inflammation as Triggering Origin of Metastasis Development. Cham, Switzerland, Springer International Publishing, 2017, pp 123-159

[25] Global Forum for Health Research & World Health Organization: The 10/90 Report on Health Research. Health San Francisco, 2002

[26] Adewole I Martin DN Williams MJ et al: Building capacity for sustainable research programmes for cancer in Africa. Nat Rev Clin Oncol 11:251-259, 2014 24614139 10.1038/nrclinonc.2014.37 PMC4403794

[27] Parker-Pope T: Cancer Funding: Does It Add Up? Available from: https://well.blogs.nytimes.com/2008/03/06/cancer-funding-does-it-add-up/, 2020

[28] Trasta A: Where does public funding for cancer research go: Allocation of research funding for cancer and COPD is not always proportional to disease burden. EMBO Rep 19:e45859, 2018 29459482 10.15252/embr.201845859 PMC5836059

[29] Spend by Research Category and Disease Site: The National Cancer Research Institute. The National Cancer Research Institute. https://www.ncri.org.uk/ncri-cancer-research-database-old/spend-by-research-category-and-disease-site/, 2020

[30] MADCaP: MADCaP. https://www.madcapnetwork.org/, 2020

[31] Prostate Cancer Transatlantic Consortium (CaPTC): EGRP/DCCPS/NCI/NIH. https://epi.grants.cancer.gov/captc/, 2020

[32] Alemayehu C Mitchell G Nikles J et al: Barriers for conducting clinical trials in developing countries- a systematic review. Int J Equity Health 17:37, 2018 29566721 10.1186/s12939-018-0748-6 PMC5863824

[33] Tertiary Education Trust Fund: http://tetfund.gov.ng/, 2020

